# Multisite gynecologic endometrioid adenocarcinomas: Can mutation profiling be used to distinguish synchronous primary cancers from metastases?

**DOI:** 10.1016/j.gore.2022.101076

**Published:** 2022-10-14

**Authors:** Dominique Barnes, Nissreen Mohammad, Lien Hoang, Michael Anglesio, Robert L. Hollis, Charlie Gourley, Heather C. Stuart, Mark S. Carey, Gavin C.E. Stuart

**Affiliations:** a Department of Obstetrics and Gynecology, University of British Columbia, Canada; b Department of Pathology, Vancouver General Hospital and the University of British Columbia, Canada; c Department of Surgery, Vancouver General Hospital, and the University of British Columbia, Canada; d The Nicola Murray Centre for Ovarian Cancer Research, Cancer Research UK Scotland Centre, MRC Institute of Genetics and Cancer, University of Edinburgh, UK

**Keywords:** Endometrioid carcinoma, Mutation analysis, Clonality, Gynecologic cancers

## Abstract

It is well recognized that some patients with endometrioid gynecological cancers have tumors arising in multiple sites (ovary, endometrium, and endometriosis) at the time of diagnosis. Molecular analysis has helped discern whether these multisite cancers represent synchronous primary tumors or alternatively metastatic disease. We present a complex case of a patient with endometrioid carcinomas arising in multiple sites. We discuss the use of mutation profiling to discern clonality and highlight how this information may inform the clinical management of such cases.

## Highlights

• Hypothesis generating case report of multisite endometrioid carcinoma.
• Molecular profiling in multisite gynecologic endometrioid adenocarcinomas; especially in pathologic and clinical uncertainty.
• Understanding multisite cancers via molecular characterization.

## 1 Introduction

Historically we have relied on clinicopathological features to make the distinction between cases of synchronous endometrioid ovarian cancers (SEOCs) versus those presenting with metastatic disease (Synchronous Ovarian and Endometrial Malignancies, 2020, Castro et al., 2000). Remarkably, the molecular evaluation of tumor tissues in cases of SEOC has established that most of these cases in fact represent metastatic disease (Wang et al., 2019). Though mutation profiling may be useful in establishing clonality, it is recognized that the interpretation of mutation profiles can be challenging due to tumor heterogeneity (Sato et al., 2000). This case illustrates clinical and molecular implications of mutation profiling as it pertains to evaluating presumed SEOC’s. Multisite cancers pose unique challenges in terms of their diagnosis, molecular characterization, and clinical management.

## 2 Case presentation

A 43-year-old woman, gravida 0, presented with abnormal vaginal bleeding and an endometrial biopsy confirmed grade 1 endometrial adenocarcinoma. Her past medical and surgical history was otherwise uncomplicated. The patient reported a slight decrease in appetite and early satiety. She endorsed oral contraception use in her 20 s. The only relevant family history included a report of a hysterectomy in the patient’s paternal grandmother for possible cancer. Initial imaging revealed a complex mass within the endometrium measuring 1.3 × 0.9 × 0.7 cm and bilateral complex adnexal masses, measuring 2 × 2 × 1 cm within the right ovary, and 5 × 5 × 4 cm on the left ovary. On initial evaluation, Ca-125 was 197 kIU/L and on subsequent testing was 620 kIU/L. The patient underwent a total abdominal hysterectomy, and bilateral salpingo-oophorectomy, omentectomy, and pelvic lymphadenectomy. Intra-operatively, scarring was noted along a portion of the sigmoid colon resulting in some folding of the colon consistent with fibrosis secondary to previous endometriosis. There were bilateral ovarian cysts with no surface disease or excrescences and the cysts were removed intact with the ovaries.

**Fig. 1:**
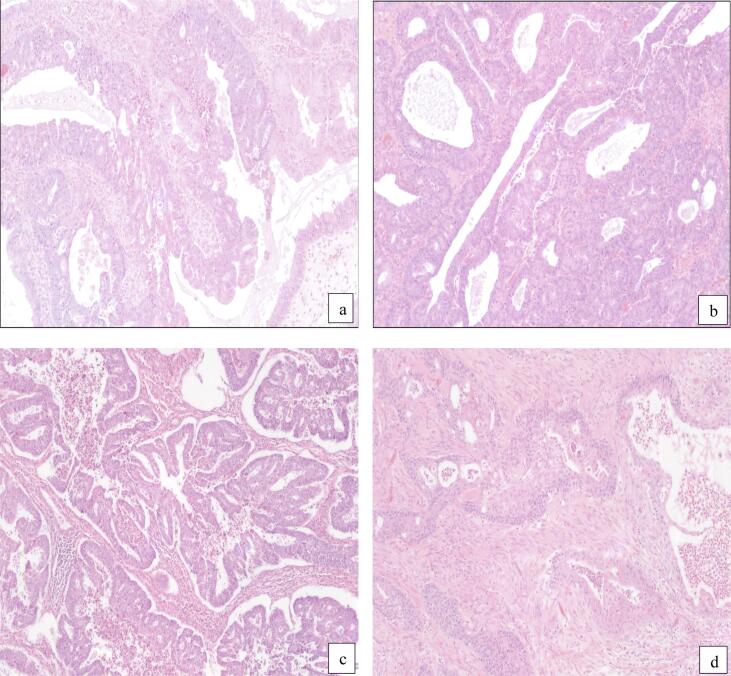
Representative hematoxylin and eosin section of the cancer sites. **Legend.** Histopathologic findings at the different sites. **a.** Left ovarian endometrioid adenocarcinoma; **b.** right ovarian endometrioid adenocarcinoma; **c.** endometrial endometrioid adenocarcinoma d. Endometrioid adenocarcinoma with extensive squamous differentiation involving the muscularis propria of the rectosigmoid colon.

Histologic evaluation showed bilateral endometrioid ovarian cancers (pT1B, G1, R0, FIGO stage IB) and a grade 1 endometrioid adenocarcinoma of the uterus (pT1a pNx, FIGO stage IA). Minimal myometrial invasion (5%) was present without any evidence of LVSI (Fig. 1). Immunohistochemistry (IHC) was performed for mismatch repair defects and p53. All tumor sites were found to have intact expression of MSH2, MSH6, MLH1, and PMS2. As well, both ovarian and endometrial cancers were p53 wild type. Based on the similar histological characteristics, and the minimal myometrial invasion, the ovarian cancers were deemed to be synchronous primary tumors and no adjuvant therapy was recommended. The patient was then advised to be followed with regular gynecological examinations. However, two months following the surgery she began to experience lower abdominal discomfort, obstipation, and a reduction in stool caliber. A CT scan showed left-sided hydronephrosis with a transition point within the left pelvis and suspected soft tissue mass effect next to this. Colonoscopy performed four months after her surgery showed a sigmoid stricture thought to be due to endometriosis with no evidence of an intrinsic lesion, though concern was raised about the potential for recurrent cancer. An attempt was made to biopsy the soft tissue abnormality, but this was unsuccessful. A left ureteric stent was inserted. Further surgery was recommended. The patient underwent laparotomy, low anterior resection with en-bloc removal of peritoneal lesion causing ureteric obstruction, left distal ureterectomy and left ureteric reimplantation with psoas hitch.

The final pathology showed a similar histologic appearance in all cancer sites (Fig. 1). Histological examination of the rectosigmoid nodule showed a FIGO grade 2 endometrioid adenocarcinoma, with lymph-vascular space invasion (LVSI) and squamous differentiation. The obstructing pelvic peritoneal nodule was positive for endometrioid adenocarcinoma arising from endometriosis. The left ureter had benign fibroadipose tissue. Twenty-three mesenteric lymph nodes were evaluated, and all were negative for malignancy. MMR testing was normal, ER was positive in all sites, POLE was negative, and the colonic mucosa showed no evidence of dysplasia. Initial post-operative PET scan showed no evidence of residual/metastatic disease. The patient was then treated with six cycles of carboplatin and paclitaxel chemotherapy and a 5-week course of external beam radiotherapy to the pelvis. Follow-up PET scan 24 months after surgery revealed an FDG avid focal liver lesion that was treated with radio-ablation. The patient remains well and on continued surveillance.

### 2.1 Pathology and mutation analysis

DNA was extracted from paraffin embedded tissue sections taken from each of the 4 cancer sites. Next-generation sequencing was used to elucidate mutation profiles for the genes and loci included in the cancer gene panel as listed in Table 1. The panel included 6 hotspots for PTEN (R130, R173, I122_M134, S170_Y188, Y225_F243, K254_K267) and 10 hotspots for PIK3CA (R88, E542, E545, Q546, D549, M1043, N1044, A1046, H1047, G1049).

**Table 1:** Hotspot Panel: CG001v4.0_Hotspot _Manifest_Panel4.0.6_20181106.tsv.Neg-Negative.Pos-Positive.

Result	Gene	Hotspot	Transcript	Result	Gene	Hotspot	Transcript
Neg	AKT1	E17	NM 001014432.1	Neg	KRAS	G12, G13, A59, O61, K117, A146	NM_004985.4
Neg	ALK	T1151, L1152, C1156, F1174, L1196, L1189, G1202, D1203, S1206, G1269	NM_004304.4	Neg	MAP2K2	Q56, K57, D67, C121, P124, P387	NM_002755.3
Neg	AR	F877, H875, L702H, S741, T878, V716, W742	NM_000044.3	Neg	MAP2K2	F57, Q60, K61, L119	NM_030662.3
Neg	BRAF	Q201, G466, F468, G469, Y472, D594, G596, L597, V600, K601	NM_004333.4	Neg	MET	Y1253, exons: 13, 14 + 25, 14–50, 14, 18	NM_001127500.2
Neg	CTNNB1	D32, S33, G34, S37, T41, S45	NM_001904.3	Neg	NRAS	G12, G13, A59, O61, K117, A146	NM_002524.4
Neg	DDR2	L239, I638, S768	NM_001014796.1	Neg	PDGFRA	D842, L839, Y849, N659, R560, E571	NM_006206.4
Neg	EGFR	S492, exons: 18, 19, 20, 21	NM_005228.3	POS	PIK3CA	R88, E542, E545, Q546, Q546, D549, M1043, N1044, A1046, H1047, G1049	NM_006218.3
Neg	ERBB2	G309, S310, L755, exons 20	NM_004448.3	Neg	POLE	Exons: 9, 10, 11, 12, 13, 14	NM_006231.3
Neg	ESR1	K303, S463, V534, P535, L536, Y537, D538	NM_001122742.1	Neg	PTCH1	W844, G1093	NM_000264.3
Neg	GNA11	O209	NM_002067.4	Neg	PTEN	R130	NM_000314.4
Neg	GNAQ	O209	NM_002067.4	Neg	RET	C634, V804, M918	NM_020975.4
Neg	GNAS	R201	NM_000516.5	Neg	ROS1	L2026, G2032	NM_002944.2
Neg	HRAS	G12, G13, O61	NM_005343.3	Neg	SMO	D473, S533, W535	NM_005631.4
Neg	IDH1	R132	NM_005896.3	Neg	TP53	Exons: 4,5,6,7,8,9	NM000547.5
Neg	IDH2	R140, R172	NM_002168.2				
Neg	KIT	T670, D816, D820, N822, Y823, A829, exons 9,11,13	NM_000222.2				

A comparison was then performed of the mutation profiles in each cancer site as outlined in Table 2. The only mutations found using the oncopanel were mutations in *PTEN* and *PIK3CA*. Two mutations (*PIK3CA*: c.3140A > G and *PTEN*: c.389G > A) were identified in both ovaries and the rectosigmoid carcinoma sample. The uterine cancer was noted to have a distinct mutation profile from the other tumor locations containing a different *PIK3CA* mutation (c.263 G > T) without the documented *PTEN* mutation found in the other sites. The endometrial tumor was sequenced twice using different blocks to confirm the findings. All tumor samples had a cellularity >=70%.

**Table 2:** Key mutations assessed by the next-generation sequencing panel.

Mutational analysis according to tumor site					
	**Gene**	**cDNA change**	**Amino Acid**	**Exon**	**Allelic ratio (%)**
Right ovary	**PTEN**	**c.389G>A (NM_000314.6)**	**R130Q**	**5**	**25.6**
	**PIK3CA**	**c.3140A>G (NM_006218.3)**	**H1047R**	**21**	**26.9**
Left ovary	**PTEN**	**c.389G>A (NM_000314.6)**	**R130Q**	**5**	**25.1**
	**PIK3CA**	**c.3140A>G (NM_006218.3)**	**H1047R**	**21**	**29.8**
Endometrium	**PIK3CA**	**c.263G>T (NM_006218.3)**	**R88L**	**2**	**32.1**
Rectosigmoid carcinoma	**PTEN**	**c.389G>A (NM_000314.6)**	**R130Q**	**5**	**28.5**
	**PIK3CA**	**c.3140A>G (NM_006218.3)**	**H1047R**	**21**	**9.8**

## 3 Discussion

Synchronous endometrial and ovarian carcinomas (SEOCs) are defined as the simultaneous presence of apparent primary cancers at the time of diagnosis. Approximately 2–9% of endometrioid uterine cancers are noted to have ovarian involvement and the endometrioid subtype is the most common histology present in these multisite cancers (Dizon and Birrer, 2016). Historically, cases of endometrioid uterine cancer with ovarian involvement were thought to represent synchronous primary cancers as they are low grade, early-stage, and usually associated with minimal myometrial invasion (Getz et al., 2013). This premise was also supported by excellent survival rates (95%) (Synchronous Ovarian and Endometrial Malignancies, 2020). It is therefore remarkable that molecular studies now confirm that almost uniformly the separate tumors in the ovaries are clonally related and represent metastatic disease from the uterus (Sato et al., 2000, Ovary Source, 2018, Reijnen et al., 2020). Using next generation sequencing (NGS), Anglesio et al. and Schultheis et al. showed that these metastatic multisite endometrioid cancers share nonsynonymous somatic mutations in several ancestral genes (Fujita et al., 2020, Schultheis et al., 2016). The TCGA analysis of endometrial cancers has showed that many of these ancestral genes (*PTEN, PIK3CA, KRAS, ARID1A, or CTNNB)* are frequently mutated (26–80%) indicating that they are likely drivers of oncogenesis (Getz et al., 2013).

In addition to traditional histopathologic assessment, molecular profiling is now routinely employed to evaluate many cancer types. We have a rapidly expanding list of molecular biomarkers that are used to improve the diagnosis and treatment of cancers. Panel sequencing is often used to characterize tumour mutation profiles and is readily available in most centers. In this case report, the uterine cancer was found to have a distinct mutation profile compared to the other sites. To better understand the application of mutation profiles various sophisticated analyses have been devised to determine clonality (Schultheis et al., 2016). However, when several mutations are shared between sites it is relatively straightforward to evaluate the probabilities of finding similar mutations in the different sites by chance. Based on data from Hollis et al., 112 endometrioid ovarian cancers were evaluated (Anglesio et al., 2016). Additional data was provided by personal communication (RH) to elucidate the mutation frequencies in this series. Overall, whole exome sequencing (WES) identified 110 nonsynonymous somatic mutations in this cohort. Specifically, H1047R mutations were only identified in 8% of samples. There were 7 PTEN mutations at the c.389 codon for a mutation frequency of 6%, however, there were only 2 cases (2%) present with the exact same mutation (R130Q). Therefore, the probability that the mutations in the two ovarian sites occurred by chance (as independent events) is 0.08*0.08*0.02*0.02, or less than 3 /1,000,000. In fact, there were no reported cases in the Hollis et al. series with the same *PTEN* and *PIK3CA* mutations together. Therefore, the sites that share these mutations there is an overwhelming likelihood that these sites are clonal in origin.

Alternatively, clonality may be assessed by determining the likelihood that shared mutations between two sites are not due to chance (Hollis et al.). Shared mutation frequency rates vary depending on the report and whether the mutation has been described in the ancestral clone or lost due to tumour heterogeneity (Reijnen et al., 2020, Anglesio et al., 2016, Hollis et al.). On average, it has been shown that only 12–46% of clonally related endometrioid ovarian cancers share the same individual mutations (Reijnen et al., 2020, Anglesio et al., 2016, Hollis et al., Hollis et al., 2021). Interestingly, neither of the two described mutations found in the ovaries or peritoneal sites in this case were found in the endometrial cancer. There are two factors however that lead us to conclude that the endometrial cancer is not clonally related. First, being that the *PTEN* and *PIK3CA* mutations were shared in 3 separate sites it is highly likely that they are ancestral. If this is true, then the uterine cancer should have the same mutations if it is clonally related. Many common ancestral mutations are drivers, and it is uncommon for driver mutations to be lost due to tumour heterogeneity. TP53 mutations in high-grade serous ovarian cancers are an example of this. The second factor is a clinical factor, as it is most uncommon for ovarian cancers to metastasize to the endometrium. Thus, it is important to note that the confirmation of clonality it may require other ancillary molecular analyses such as mutation signatures, copy number, and LOH (Hollis et al., Hollis et al., 2021). Mutation profiles comparisons between different tumor sites may not provide enough information to establish clonality and these additional analyses could be considered in circumstances where the establishment of clonality will change clinical management.

It is evident that next generation sequencing will play a greater role in clinical decision-making for the management of endometrioid ovarian cancers. Molecular testing using a combination of sequencing and hormone expression can define prognosis in endometrioid ovarian cancers and may also have predictive value (Hollis et al., Hollis et al., 2021). Based on this case report we cannot recommend routine sequencing of SEOC cases; however, it may be useful in selected cases where there is pathological or clinical diagnostic uncertainty. The confirmation of metastatic grade I endometrioid cancers in such cases may spare patients unnecessary and costly adjuvant treatment. With the declining cost of next generation sequencing, mutational profiling of these cases may be cost-effective. Adjuvant treatment may be costly but the declining expense of next generation sequencing; mutational profiling may be cost effective for these selective cases.

In cases of multisite endometrioid cancers, the classical clinical and pathological criteria are unable to accurately distinguishing (SEOCs) from metastatic disease (Wang et al., 2019, Sato et al., 2000, Dizon and Birrer, 2016). Mutation profiles may be informative particularly when multiple mutations are shared between sites. As we demonstrate, clonality can be determined with confidence in this setting. It is interesting and paradoxical that patients presenting with low-grade endometrioid carcinomas metastatic to ovary from endometrium have an excellent prognosis. In fact, this case represents an exception with strong evidence that the uterine and ovarian sites represent SEOCs.

## 4 Conclusion

Multisite endometrioid cancers represent a unique and interesting clinical challenge. In these cases, mutation profiling may be very helpful for determining clonality, particularly when more than one mutation is shared between sites. Due to the impact of tumour heterogeneity, when different tumour sites do not share the same mutations other types of molecular studies may useful to establish clonality. This case illustrates the role of mutation and molecular profiling in cases of SEOC and this information may be important for evaluating prognosis and future treatment recommendations.

## Declaration of Competing Interest

The authors declare that they have no known competing financial interests or personal relationships that could have appeared to influence the work reported in this paper.
